# Evaluation of Clove Extract for Drug Therapy of Ciliate Infection in Coral (*Goniopora columna*)

**DOI:** 10.3390/biology11020280

**Published:** 2022-02-10

**Authors:** Tah-Wei Chu, Chiu-Min Cheng, Yu-Rong Cheng, Cheng-Di Dong, Chih-Hung Chuang, Chih-Hung Pan, Wei-Ting Sun, De-Sing Ding

**Affiliations:** 1 Department and Graduate Institute of Aquaculture, National Kaohsiung University of Science and Technology, Kaohsiung 811, Taiwan; twchu@nkust.edu.tw (T.-W.C.); cmcheng@nkust.edu.tw (C.-M.C.); pc116605t@yahoo.com.tw (C.-H.P.); 1051537103@nkust.edu.tw (W.-T.S.); 2 Department of Fisheries Production and Management, National Kaohsiung University of Science and Technology, No. 142, Haijhuan Rd., Nanzih District, Kaohsiung 811, Taiwan; yrcheng@nkust.edu.tw; 3 Department of Marine Environmental Engineering, National Kaohsiung University of Science and Technology, Kaohsiung 811, Taiwan; cddong@nkust.edu.tw; 4 Department of Medical Laboratory Science and Biotechnology, College of Health Sciences, Kaohsiung Medical University, Kaohsiung 807, Taiwan; a4132600@gmail.com

**Keywords:** clove, ciliate, *Goniopora columna*, antioxidant enzymes, treatment

## Abstract

**Simple Summary:**

In recent years, studies have found that coral infectious diseases are gradually spreading. Ciliate disease poses a serious threat to corals, and infected corals will fester and die within a short period of time. Clove is a traditional Chinese medicine. In this study, Clove extract was used to evaluate the treatment of ciliate diseases to achieve safety and to reduce the stress response of corals. Studies have shown that 1500 ppm clove extract can effectively treat ciliate parasitism, and does not affect coral zooxanthellae, chlorophyll a, or stress response. This extract has been successfully applied to a Taiwan coral king coral farm, which will have great significance for large-scale coral aquaculture.

**Abstract:**

In recent years, ciliate infections have caused serious casualties to corals in the ocean. Infected corals die within a short period of time, which not only poses a threat to wild coral reefs, but also has a major impact on large scale aquaculture of coral. Clove is a kind of Chinese medicine with antifungal, antibacterial, antiviral, insecticidal, and other functions. Clove is a natural product. If it can be used in the treatment of coral ciliates, it will reduce this threat to the environment. The clove extract was diluted with sterile seawater to 500 ppm, 1500 ppm, 2500 ppm, 5000 ppm, 7500 ppm, and 10,000 ppm to carry out virulence test on ciliates. The results show that the LC_50_ value is 1500 ppm, which can cause the death of ciliates in 10 min without causing significant changes in *G. columna* SOD, CAT, chlorophyll a, and zooxanthellae. In addition, observation of tissue slices revealed that no ciliates and vacuum were found in the *G. columna* tissue after 10 min of medicated bathing. In summary, 1500 ppm of clove extract can be used for the treatment of coral ciliates.

## 1. Introduction

Global climate change, sedimentation, nutrient enrichment, and ocean warming have caused some disease outbreaks in coral, leading to death [1,2]. Bourne et al. (2008) [3] found that coral infectious diseases have gradually continued to spread in recent years. At present, ciliates have been found to parasitize corals and cause large areas of bleaching and death in the Caribbean Sea, the Great Barrier Reef, and the Pacific Ocean, which are important coral reef areas in the world [3,4]. To date, the diseases that have been found to cause coral infections include white band disease (WBD), white plague (BP), brown jelly syndrome (BJS), brown band syndrome (BrB), skeletal eroding band (SEB), etc., [5,6,7]. The BJS, BrB, and WS diseases are all caused by ciliate infection, and the infected coral tissues become pathological and produce a characteristic jelly-like substance [8,9]. Coral infection with ciliate disease will cause tissue bleaching and ulceration at the initial stage, which has a high mortality rate [10]. In addition, due to the booming global aquarium ornamental trade in recent years, corals infected with ciliates may also be transported to aquariums around the world [10]. This may cause the spread of ciliate infections to accelerate, and effective prevention and treatment will curb the threat of ciliates to corals. According to previous studies, it has been found that folliculinid ciliate (*Halofolliculina corallasia*) infection can cause diseased coral tissues to produce speckled black bands [6]. The BrB infection has caused serious casualties on the three corals of the Great Barrier Reef, Acroporidae, Pocilloporidae, and Faviidae [11]. *Halofolliculina* sp. can invade and parasitize through damaged coral tissues, causing a coral to fester and die [12]. Therefore, if the coral is bitten by a fish or *Acanthaster planci*, it may cause *Halofolliculina* sp. invasive infection [13]. This has a considerable impact on the sustainable development of coral reefs and the development of large-scale coral aquaculture. *Porpostoma guamensis* will swallow coral tissues, zooxanthellae, and eggs, causing brown bands to form on the coral [14]. Coral ciliate infection is one of the most important diseases affecting the survival of coral reefs. Corals infected with ciliates can undergo rapid tissue decay and death [14,15]. There are few studies on the pathogenic potential and treatment of corals caused by ciliate disease. Our recent research has found that *Philaster lucinda* is a potential parasite of corals. It feeds on coral tissues and zooxanthellae. After infection, it will cause corals to die quickly. Its treatment and prevention are very important [10]. Sweet and Séré, (2016) found that corals infected with ciliates (*P. lucinda*) die within 24 h [16]. Page and Willis found that the *Acropora muricata* tissue ulceration rate after *P. lucinda* infection is ~2 mm/day, which means the coral will die shortly after infection [17]. Coral infection by *P. lucinda* will cause coral bleaching and death within 72 h [10]. Previous research mainly focused on the investigation of diseases in wild corals. Therefore, effective prevention and treatment of coral ciliate diseases is an important area of research.

Clove (*Syzygium aromaticum*) is a Chinese medicine, a terrestrial plant, with a natural fragrance, and it is also a spice [18,19]. It has antiseptic, antifungal, antibacterial, antiviral, and anticancer activities [18]. Toxic at high concentrations, it will cause tissue damage and hepatotoxicity [20]. In addition, the heterogeneous distillate made from crushed clove, commonly known as clove oil, has the function of anesthesia and treatment of toothache [21]. Clove oil is used to sedate fish during transport or surgery [22]. The active substance contained in clove oil is eugenol (4-allyl-2-methoxyphenol), which accounts for about 70–95%, and the remaining 5–30% is made up of eugenol acetate and kariofilen-5 [18,21,23]. In aquaculture applications, clove oil is often used as an anesthetic or as an alternative to cyanide for fishing coral reef fish [18,24]. Previous research indicated that limited amounts of clove oil solution are unlikely to harm *Pocillopora damicornis* [19]. At present, clove oil is used to kill ciliates in the purification and cultivation of microalgae [25]. Therefore, this study explores whether natural clove extract can be used to kill ciliates without harming corals.

In this experiment, the antioxidant enzymes superoxide dismutase (SOD) and catalase (CAT) were used to evaluate the stress response of corals under treatment. The biological metabolism requires O_2_, and organisms produce free radicals and reactive oxygen species (ROS). The stress response can lead to a gradual increase in coral ROS, causing coral bleaching or death [26,27]. Disease and environmental stress can induce ROS elevation, causing oxidative damage in the coral–zooxanthellae symbiotic system [28,29,30,31]. Corals respond to stress by producing antioxidant enzymes (SOD and CAT) to suppress ROS. The defense mechanism of cells against the formation of oxygen radicals involves enzymes such as SOD and CAT to act in concert to inactivate superoxide radicals and hydrogen peroxide. This prevents the formation of the most reactive form of ROS, the hydroxyl radical, and subsequent cellular damage [10,31]. SOD catalyzes the dismutation of superoxide into oxygen and hydrogen peroxide, and CAT is responsible for inactivating hydrogen peroxide into water and oxygen [10]. These enzymes are responsible for detoxifying ROS, and their elevated activities indirectly indicate an increased production of ROS in corals as a result of disease or environmental stresses such as poor water quality, UV radiation, irradiance, and temperature [28]. Cheng et al., (2021) [10] pointed out that *G. columna* infections with ciliates can cause significant changes in superoxide dismutase and catalase in the body. Therefore, this study uses superoxide dismutase and catalase changes as the stress response of *P. lucinda* infection and clove extract treatment of coral cells.

This study hopes to use safer and natural ingredients to treat ciliate diseases. If corals can be soaked in clove extract for a long time without damaging the coral tissues, it will be of very helpful for the sustainability of coral reefs and large-scale aquaculture of *G. columna*. The ciliates (*P. lucinda*) in this experiment were purified and cultured from coral. After infecting the *G. columna* with ciliates, clove extract was used for treatment to observe changes in *G. columna* morphology, chlorophyll a content, number of zooxanthellae, tissue and survival, and to detect antioxidant enzyme activity (superoxide dismutase and catalase) to determine the stress response of the coral after treatment, in order to evaluate whether the clove extract can be used as a treatment for coral ciliates.

## 2. Materials and Methods

### 2.1. Experiment 1, Evaluation of Toxicity and Coral Drug Tolerance of Clove Extract on Coral Ciliates Disease

#### 2.1.1. Purification and Identification of Ciliates

The ciliates used in this experiment were from infected *G. columna* coral for pure culture. Refer to [10,16] for the identification of ciliates. Total DNA of ciliates was sampled from infected corals using a genomic DNA isolation kit (Protech Technology Enterprise Co., Ltd., Taipei, Taiwan). Amplification of universal eukaryotic 18S rRNA gene was performed using the primers 4617f and 4618r [16]. The operation method of PCR was as follows: the sample was preheated at 95 °C for 3 min for 35 cycles (95 °C; 30 s, 55 °C; 30 s, and 72 °C; 1 min), then 72 °C; 5 min. Next, 1 uL of the PCR product was used for the nested PCR with the ciliate-specific primers 384f-cil and 1147r-cil [16]; the annealing temperature of the PCR was 60 °C. The PCR product was cloned into pCR-Blunt (Zero Blunt cloning kit; Invitrogen) and sequenced by a biotech company. Gene sequence alignment was performed using the Basic Local Alignment Search Tool. The pure ciliates were a pure culture in 50 mL sterile saltwater and fed with coral polyps daily. We used a microscope (Leica DM500, 400×) to observe zooxanthellae in the ciliates every 24 h. If the zooxanthellae were digested in the body, *G. columna* polyps were added. We changed the water once a week during the cultivation period. Please refer to Table 1 for the water quality parameters. After 48 h of incubation, we used a microscope (Leica DM500) at 100× magnification to calculate the number of ciliates using a hemocytometer [10].

#### 2.1.2. Clove Extract

The extraction method of this experiment refers to Cortés-Rojas et al., 2014. After grinding 50 g of dried clove buds, we put them in an Erlenmeyer flask and used 70% (*v*/*v*) ethanol for 48 h of extraction. The ratio of plant to solvent was 1/10 (*w*/*v*). The extractive solution was concentrated in a rotary evaporator using a vacuum pressure of 600 mmHg at a maximum temperature of 55 °C. After the extraction was completed, the extract was freeze-dried (Boyer et al., 2009) [32]. We found that using ethanol as a solvent in Clove oil caused coral bleaching. Therefore, we used sterile seawater as a solvent. The use of seawater dilution can prevent errors caused by osmotic pressure. The concentrations of clove extract were 500, 1500, 2500, 5000, 7500, and 10,000 ppm, no medication was used on control group C.

#### 2.1.3. Coral Samples

*G. columna* was taken from Taiwan Coral King Coral Farm of Taiwan (CITES legal coral farm, CITES number FTS507W0153796). The coral culture method refers to [33], and a total of hundred colonies of the corals were kept in a tank (60 × 35 × 30 cm^3^) with a recirculating filtered seawater system. After two months of self-repair and acclimation, healthy *G. columna* were cut into 5 polyps per colony, and then stuck on the rough cornerstone with coral glue; each group contained ten colonies. Each experiment was repeated in triplicate, and each group contained thirty colonies (*n* = 30). For the water quality parameters for farming corals, please refer to Table 1.

#### 2.1.4. Ciliate Toxicity Test

In this experiment, refer to [10,34]. Cultured coral ciliates were microscopically observed for viability and health and counted using hemocytomete. The density of ciliates used in this experiment was 1 cell mL^–1^. Chen et al. (2021) [10] found that, at this density, infection can cause *G. columna* to fester and die 72 h after infection. Therefore, the clove extract was diluted with sterile saltwater to 500, 1500, 2500, 5000, 7500, and 10,000 ppm to conduct toxicity tests on the *P. lucinda*, the death time and number of ciliates were recorded, and the experiment ended when the ciliates completely died. Referring to the method of Cheng et al. [10], we use the LC_50_ value to represent the 50% lethal concentration of ciliates.

#### 2.1.5. Evaluation of Coral’s Drug Tolerance to Clove Extract

In this experiment, we refer to Ding et al. [33] for *G. columna* aquaculture methods. The specimens were maintained and fed in a recirculating filtered tank (60 × 35 × 30 cm^3^) with a saltwater system, using a beaker (500 mL) filled with sterile saltwater for the experimental grouping. The 500 mL beaker was put in a recirculation filtration and constant temperature tank to ensure consistent water conditions. After observation for 2 h, we waited for the coral polyps to fully extend before starting the test. The ciliate toxicity test results showed that 500 ppm would not cause the death of ciliates, so the tested concentrations of clove extract in this experiment were 1500 ppm 2500, 5000, 7500 and 10,000 ppm. Samples were taken after 10 min and 24 h, respectively, for zooxanthellae, chlorophyll a, polyp length, survival rate and two antioxidant enzyme (SOD and CAT) activity tests to assess whether long-term dipping would cause a coral stress response.

#### 2.1.6. Analysis of Two Antioxidant Enzymes

The detection method of this experiment refers to [10,35], the *G. columna* tissue was homogenized and we added nine times the volume of ice-cold extraction buffer to dissolve (20 mM phosphate buffer, 0.1% (*v*/*v*) Triton X-100; pH 7.4, 1 mM EDTA). The coral crude extract was sonicated at low temperature (3 s each, 5 times), centrifuged (12,000× *g*, 5 min, 4 °C) and used as a coral tissue solution for enzyme and protein determination.

#### 2.1.7. Superoxide Dismutase and Catalase Detection

The detection method of SDO refers to Higuchi et al. (2008) [31] and Chen et al. (2021) [10], and we used assayed spectrophotometrics to measure it. Each treatment group first used bovine erythrocytic (Sigma) to prepare the active standard. All measurements were performed at a constant temperature of 25 °C. Protein content was measured using the Bradford assay [36]. CAT detection refers to Main, Ross, and Bielmyer. (2010) [37] and Chen et al. (2021) [10], using Sigma commercial reagents. First, we added the coral tissue solution (15 mL) to the tube, added reagents and centrifuged, and took the supernatant as a test sample. Finally, absorbance was measured using OD 520 nm. We refer to the calculation formula of Cheng et al. (2021) [10] for CAT calculation.

#### 2.1.8. Protein Concentrations

The coral specimens were homogenated and sonicated, and protein concentrations were measured using a commercially available reagent Bradford protein assay kit (Amresco, Solon, OH, USA), with bovine serum albumin serving as the protein standard.

#### 2.1.9. Analysis of Zooxanthellar Density and Chlorophyll a

This experiment was performed according to the methods of Levy et al. [38] and Cheng et al. [10]. First, we homogenized the coral tissue and then used the blood cell counter to calculate the zooxanthellae. The density of zooxanthellae represented the content of each polyp. The detection method of chlorophyll a was based on the methods of Levy et al. [38] and Titlyanov et al. 2001 [39]. We took 0.5 g of coral tissue for homogenization, added 10 mL (90% acetone), and let it stand for 24 h under completely dark conditions at a constant temperature of 4 °C. Finally, measurements were made with absorbance OD 630 and 664 nm. The concentration calculation was carried out with reference to the calculation formula of Jeffrey and Humphrey [40].

### 2.2. Experiment 2, Clove Extract for Treatment of Coral Ciliates Disease

#### 2.2.1. Ciliate Disease Treatment Test

In experiment 1, it was known that the LC_50_ of clove extract against ciliates is 1500 ppm and that it can cause complete death within 10 min. Therefore, in the study of the ciliate disease treatment and drug tolerance of coral, 1500, 2500, 5000, 7500 and 10,000 ppm were selected as test concentrations. No medication was used on control group C. This experiment refers to Cheng et al. (2021) [10]. After the corals were infected by ciliates, they were dipped in different clove extract concentrations for 10 min, and then replaced in sterilized seawater. After 72 h, we referred to Levy et al. (2003) [38] and Chen et al. (2021) [10] for the method to judge the change in coral morphology, and the survival, chlorophyll a, zooxanthellae number, antioxidant enzymes superoxide dismutase and catalase were measured to evaluate the effect of drug treatment on the coral. After the experiment, the seawater in the beaker was centrifuged, the supernatant liquid was removed, and the ciliates in the saltwater were calculated using a hemocytometer.

#### 2.2.2. Histology Observation

After the treatment experiment was over, *G. columna* tissues were fixed with 4% formalin for 48 h, then soaked with 5% formic acid until the bones were completely dissolved, and then dehydrated with alcohol for paraffin embedding and tissue sectioning. Routine tests were carried out for histology, tissue sectioning, and staining with hematoxylin and eosin (H and E) [14,41].

### 2.3. Statistical Analysis

Data were obtained from two independent experiments, and the final results were presented as the mean ± standard deviation (SD). One-way analysis of variance and Duncan’s multiple range test were used to determine the statistical significance for survival, SOD, CAT of *G. columna*, as well as its chlorophyll a and zooxanthellar density content. A *p*-value of <0.05 was considered significant. All statistical analyses were performed using IBM SPSS statistics 20.

## 3. Results

### 3.1. Experiment 1, Evaluation of Toxicity and Coral Drug Tolerance of Clove Extract on Coral Ciliates Disease

#### 3.1.1. Toxicity Test of Clove Extract on Ciliates

The toxicity test results for the clove extract on *P. lucinda* are shown in Table 2. At a poisoning time of 600 s, the 500 ppm concentration of clove extract did not cause death to ciliates. When the concentration was above 1500 ppm, death began to occur after 300 s (61.67 ± 5.57%), and the pathogens were completely dead by 420 s. With 2500 ppm, they began to die at 240 s (58.33 ± 2.89%), and the mortality rate reached 100% at 360 s. Deaths occurred within 60 s for the 5000, 7500, and 10,000 ppm experiments. The mortality rates were 80.33 ± 1.53%, 75.33 ± 5.51%, and 38.33 ± 7.64%, respectively. The 100% mortality of the three treatment groups all occurred within 300 s. In conclusion, the LC_50_ was 1520 ppm for clove extract, which can cause *P. lucinda* to die after 420 s of treatment.

#### 3.1.2. The Effect of Clove Extract on *G. columna* Stress Response

**Figure 1:**
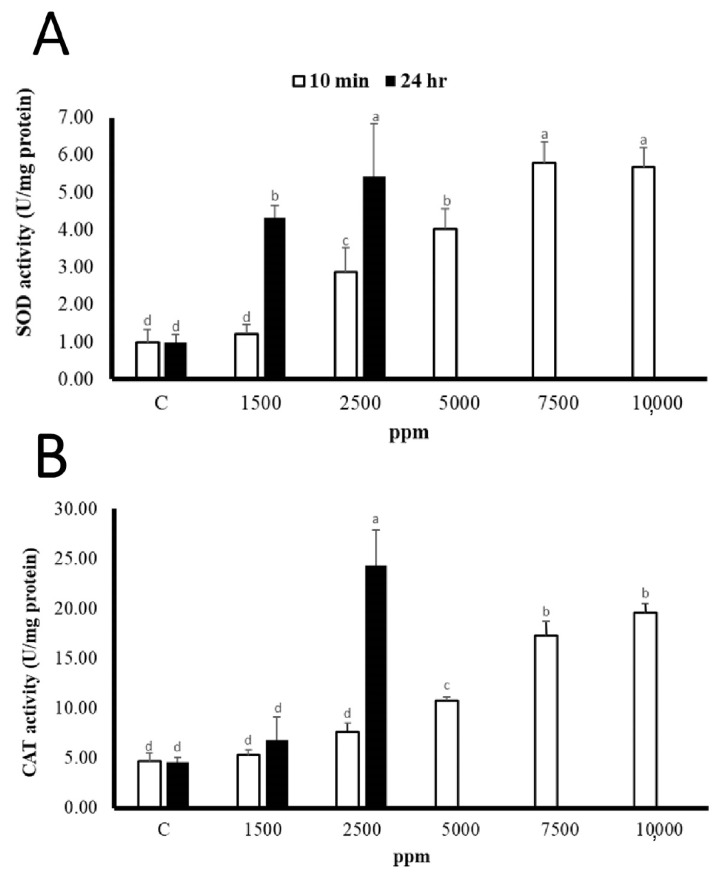
Clove extract response to coral stress. Different letters indicate significant differences among groups (*p* < 0.05). The values are expressed as means ± SDs (*n* = 30 colonies): (**A**) SOD; (**B**) CAT. The treatment group above 5000 ppm could not be detected due to the death of the corals.

The tolerance of corals to clove extracts was evaluated based on the stress response of corals to clove extracts. The results of Figure 1 show that after 10 min of stress response, coral SOD activity increased significantly. The highest SOD activities, at 7500 and 10,000 ppm were 5.78 ± 0.56 U/mg protein and 5.67 ± 0.52 U/mg protein, respectively, and the lowest of the treatment group was at 1500 ppm, which was 1.21 ± 0.34 U/mg protein. The difference in SOD activity between the two was about 4.37 times. There was no significant difference between the SOD activity of C and 1500 ppm for 10 min of dipping. In addition, long-term dipping for 24 h showed that the SOD activity at 1500 ppm and 2500 ppm differed by 1.25 times.

The results of the CAT are shown in Figure 1. There was no significant difference between C and 1500 and 2500 ppm in 10 min of treatment. The CAT activity of the 5000 ppm treatment group began to increase significantly, and at 10,000 ppm the CAT activity reached the highest at 19.57 ± 0.90 U/mg protein, which was 3.71 times higher than that of 1500 ppm. The 24-h treatment results show that there was no significant difference between C and 1500 ppm, and that 2500 ppm was 3.58 times higher than 1500 ppm. In the treatment groups above 5000 ppm, the corals were dead, and the activity of SOD and CAT could not be detected (Table 3). Therefore, the higher the concentration or the longer the dipping time, the more significant the stress response of the corals.

#### 3.1.3. Coral Drug Tolerance Assessment

The drug tolerance evaluation of coral after clove extract treatment for 10 min and 24 h of dipping is shown in Table 3. After 10 min of treatment of *G. columna*, the survival rate of each treatment group was 100%. The polyp length of each treatment group was significantly shortened, the largest difference was the treatment group above 5000 ppm, whose polyps were shortened to 0.10 cm. Further, polyps in the 1500 ppm and 2500 ppm treatment conditions were 1.05 and 1.61 times shorter than the C group. The zooxanthellae of group C was 5.10 ± 2.54 cells × 10^7^ m^−2^, which was nonsignificant compared with each treatment group. Chlorophyll a in each treatment group was also nonsignificant.

After a long period of 24 h of treatment, the survival rate of each treatment group was significantly reduced, and the survival rate of each group above 5000 ppm treatment was 0%. There was no significant difference between the C and 1500 ppm polyp length, while the other treatment groups had significant polyp shortening, which was reduced to 0.00 cm at 7500 ppm. The content of zooxanthellae and chlorophyll a in the corals showed that there was no significant difference between C and 1500 ppm, and there was a significant decrease in the other treatment groups. In short, in addition to the concentration of clove extract that affects the drug tolerance of corals, dipping time can also cause corals to die.

### 3.2. Experiment 2, Clove Extract for Treatment of Coral Ciliates Disease

#### 3.2.1. Drug Therapy Evaluation

According to the experimental results, Table 4 shows. *P. lucinda* was infected with *G. columna*, and after dipping with different concentrations of clove extract for 10 min, after 72 h it was found that the survival rate of control group C was only 11.35 ± 5.73%, and the survival rate of other clove extract concentration groups was 100%. By observing the morphological differences of coral polyps after infection by ciliates, the length of coral polyps was 0.00 cm, and the polyps were completely retracted into the corallites. The polyp length of each treatment group was the longest, 1.00 ± 0.20 cm, at 1500 ppm treatment, which was 10 times worse than 10,000 ppm. The zooxanthellae density was the highest in the treatment group of 1500–5000 ppm, which was 20.32 times higher than that of C. Chlorophyll a also had the highest content in the 1500–5000 ppm treatment group, which was 8.52 times higher than that of C.

#### 3.2.2. Evaluation of the Treatment’s Response to *G. columna* Stress

**Figure 2:**
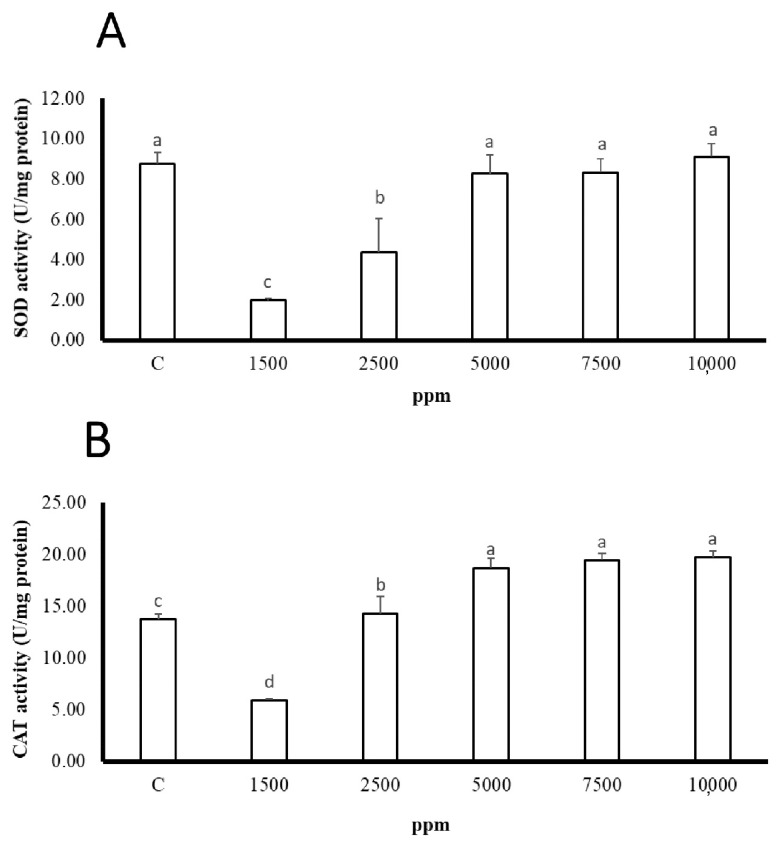
Changes to coral antioxidant enzymes activity during ciliate disease treatment. C: infected; clove extract was not used. Different letters indicate significant differences among groups (*p* < 0.05). Values are expressed as means ± SDs (*n* = 30 colonies): (**A**) SOD, (**B**) CAT.

After the *P. lucinda* were infected, we used the clove extract to dip for 10 min, and after 72 h, the superoxide dismutase and catalase activities in the *G. columna* were as shown in Figure 2. The activity of 1.98 ± 0.09 U/mg protein at 1500 ppm was low, and the activity of SOD reached 8.27 ± 0.93 U/mg protein at 5000 ppm, which was the highest. The difference between the two was 4.18 times. The SOD activities of the 7500–10,000 ppm groups were 8.33 ± 0.67 U/mg protein and 9.12 ± 0.64 U/mg protein, respectively. Compared with the 1500 ppm group, it increased by 4.21 times and 4.61 times, respectively. CTA activity was lower at 1500 ppm, and CAT activity was 2.31 times lower than that of infected C. Furthermore, the 5000, 7000, and 10,000 ppm treatment group CAT activity increased by 3.15, 3.27, and 3.31 times compared with 1500 ppm group, respectively. Therefore, the higher the concentration of clove extract, the greater the impact on the stress response of *G. columna*.

#### 3.2.3. The Impact to Coral Tissue

**Figure 3:**
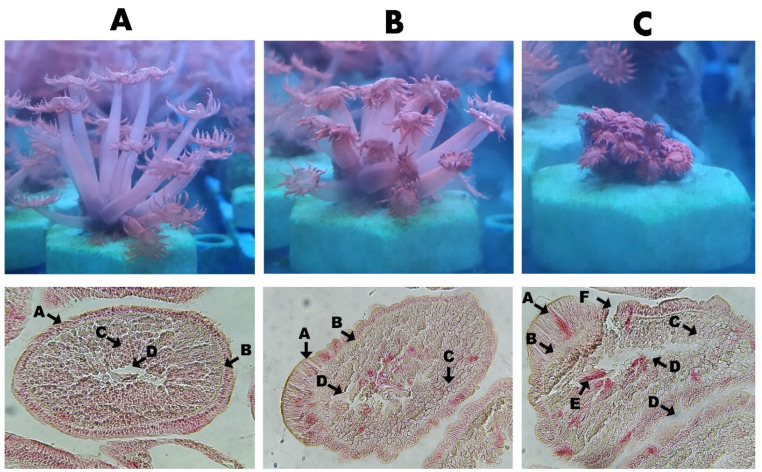
Image and tissue section observation of *G. columna* infected with ciliates and treatment with clove extract. (**A**) No infection. Polyps fully stretched, no tissue damage (A, ectoderm; B, mesoglea; C, endoderm and zooxanthellae; D, stomach). (**B**): After being infected with ciliates, dipping was performed with 1500 ppm clove extract for 10 min. Coral polyps atrophied slightly, and the mesoglea and endoderm were not infected by ciliates. The endoderm contains the corpses left by the death of the ciliates (A, ectoderm; B, mesoglea; C, endoderm and zooxanthellae; D, stomach). (**C**): C group. Ciliates parasitize the endoderm and swallow zooxanthellae, causing serious damage to cells and tissues (A, ectoderm; B, mesoglea; C, endoderm and zooxanthellae; D, damaged tissue will produce a vacuum; E, *P. Lucinda*; F, ciliates infection causes damage to the tissues of the ectoderm, mesoglea and endoderm).

The tissue changes after treatment with clove extract are shown in Figure 3. This study was based on Work and Meteyer (2014) [42] to judge the damage to coral tissue. The coral structure consisted of the simple structures of ectoderm, mesoglea, and endoderm. Ciliates will cause serious damage to the endoderm after infecting *G. columna*, and the damaged tissue will produce a vacuum. From the tissue slice, it can be observed that the infected endoderm tissue contained ciliates. When the ciliates parasitize the endoderm, they swallow the coral’s zooxanthellae and tissues, resulting in tissue damage and empty vacuola. Healthy tissues do not contain a vacuum. After using the clove extract at 1500 ppm and dipping for 10 min, no ciliates were found in the tissue, and no tissue damage was observed.

## 4. Discussion

Few previous studies have explored coral diseases and treatments, and the treatment of coral diseases is quite important for marine aquarium and large-scale coral aquaculture [10]. According to 18S rRNA and morphology observations, the purified ciliates in this experiment are *P. Lucinda*. Our discovery of ciliate GenBank matches up to 100% with the P. lucinda DNA sequence found in the Caribbean Sea [10,16]. This kind of ciliate infects *G. columna* and *Euphyllia glabrescens*, and causes coral festering and death [10]. This poses a serious threat to wild coral reefs and large-scale coral aquaculture.

Although we recently published that KCl or H_2_O_2_ can be used to achieve effective treatment of ciliate infections, it was found that KCl or H_2_O_2_ stimulation reduced the zooxanthellae and affected the color of corals [10]. After using H_2_O_2_ treatment, the length of polyps was shortened by 19.30 times, and the number of zooxanthellae also decreased significantly [10]. Therefore, if a milder natural medicine can be found for treatment, it will alleviate the corals’ stress response.

This study found that 1500 ppm of clove extract caused the death of ciliates in 300 s, and the higher the concentration, the faster the death rate. When the concentration reached 5000 ppm, the ciliates died completely within 60 s. Previous studies have indicated that clove oil will not affect the coral color or photosynthetic efficiency at 0.5 ppt, but it will kill corals immediately at 50 ppt [19]. In addition, clove oil contains eugenol and β-caryophyllene, which can be used to remove ciliates during the purification and cultivation of microalgae [25]. Clove oil can also be used as attract-and-kill treatment against *Bactrocera dorsalis* [43]. Therefore, according to the experimental results, it is feasible to use clove extract for the treatment of ciliate disease in coral.

According to Frisch et al. (2007), the use of a clove oil–ethanol mixture (10% clove oil) can cause *P. damicornis* to albinize and die. Compared with the control group without the addition of ethanol or lower exposure (10 mL), no coral bleaching or death was found [19]. This indicates that the mixture produced by the interaction of clove oil and ethanol was more toxic to corals [32]. The results of this experiment show that after dipping in 5000–10,000 ppm of clove extract for 10 min, the activities of SOD and CAT both increased significantly, and the higher the concentration, the higher the activity of antioxidant enzymes, and the shorter the polyp length of each treatment group, but there was no significant difference between chlorophyll a and zooxanthellae, and no coral bleaching or death was found. When the dipping time was extended to 24 h, the corals in the treatment groups above 5000 ppm died. Therefore, long-term dipping in clove extract will cause *G. columna* death, while using 1500 ppm for a short time of 10 min was effective. Soaking will not cause coral casualties. The polyp length, CAT, and SOD activities had no significant changes compared with the control group. Previous studies found that the superoxide dismutase and catalase activities of KCl 1.5% treatment were 3.51 times lower than those of H_2_O_2_ 1.5%, but the SOD and CAT activities of the KCl 1.5% treatment were at least 1 time higher than those of the control group without additions [10]. The results of this study showed that there was no significant change in SOD and CAT activity compared with the control group after 10 min of treatment with clove extract at 1500 ppm. Therefore, clove extract is more suitable for the treatment of coral ciliate disease than KCl and H_2_O_2_.

A previous study found that, after five weeks of cultivation using 14% clove oil in a lagoon for 3 days, the growth of *Acropora striata*, *Pocillopora verrucosa,* and *Porites australiensis* decreased by an average of 30–40%, and the chance of coral bleaching increased by 20–80% [32]. Exposing *P. verrucosa* in Scleractinaire to a 50% clove oil–ethanol solution for 1 min will cause 100% bleaching [44]. In addition, clove oil often uses ethanol as a solvent. If clove oil is dissolved in ethanol, it will cause a more serious bleaching of corals [32]. This may be because ethanol increases the solubility of clove oil in seawater or produces toxic compounds that will harm corals [32]. This may increase the permeability of clove oil in coral tissues. Therefore, this study used sterile seawater as the solvent for the clove extract, and did not use ethanol. Soaking at 5000 ppm for 24 h will cause coral death, while soaking for 10 min will not cause coral death and can effectively treat ciliate disease. Cheng et al. (2021) [10] found that the use of KCl or H_2_O_2_ for ciliate disease treatment reduced the number of zooxanthellae in the coral endoderm, resulting in a dull or white coral color. Although it can be restored after a period of time, it will affect the coral’s viewing value for a short time. After 10 min of a one-time treatment with clove extract 1500 ppm, the coral configuration, zooxanthellae, and chlorophyll a were not significantly changed after one week. Therefore, clove extract can only be used as a short-term medicinal bath or in simple use. It will not cause a stress response in the coral or cause configuration changes within 10 min of soaking, but long-term soaking will cause the coral to die.

## 5. Conclusions

According to the experimental results, clove extract can be used in the treatment of coral ciliates. According to LC_50_, antioxidant enzyme activity and morphological observations after treatment, it is estimated that the appropriate concentration is about 1500 ppm, and a medicated bath can achieve effective prevention and treatment within 10 min. This concentration will not cause the coral to produce a strong stress response, nor will it cause coral bleaching, death, or color change. It can be used in marine aquarium and coral large-scale aquaculture. This technology has been practically applied in the Taiwan Coral King coral farm for the medication of drug-sensitive corals. It is hoped that this research can contribute to large-scale coral aquaculture and the sustainable development of wild coral reefs.

## Figures and Tables

**Table 1:** The water quality conditions in the study.

Water Quality Conditions	Ciliates	Coral
Temperature (°C)	26.0 ± 0.5	26.0 ± 0.5
pH	8.0 ± 0.5	8.0 ± 0.5
Dissolved oxygen (ppm)	6.00 ± 0.05	6.00 ± 0.05
Nitrous acid (ppm)	0.01 ± 0.01	0.01 ± 0.01
Nitric acid (ppm)	0.05 ± 0.02	0.02 ± 0.01
Calcium (ppm)	425 ± 40.12	455 ± 10.32
Magnesium (ppm)	1324 ± 63.21	1360 ± 52.21
Ammonia nitrogen (ppm)	0.01 ± 0.05	0.01 ± 0.05
Phosphate (ppm)	0.01 ± 0.01	0.01 ± 0.01

Values are expressed as means ± SDs (*n* = 3).

**Table 2:** Inhibitory concentrations of clove extract on ciliates.

	Survival Rate% (Mean ± SD)						
Sec	C	500 ppm	1500 ppm	2500 ppm	5000 ppm	7500 ppm	10,000 ppm
60	100 ± 0.00	100 ± 0.00	100 ± 0.00	100 ± 0.00	80.33 ± 1.53	75.33 ± 5.51	38.33 ± 7.64
120	100 ± 0.00	100 ± 0.00	100 ± 0.00	100 ± 0.00	54.00 ± 4.36	50.00 ± 2.65	32.33 ± 7.51
180	100 ± 0.00	100 ± 0.00	100 ± 0.00	100 ± 0.00	40.00 ± 3.61	40.67 ± 1.53	13.33 ± 1.53
240	100 ± 0.00	100 ± 0.00	100 ± 0.00	58.33 ± 2.89	15.33 ± 1.53	11.00 ± 1.00	6.33 ± 2.52
300	100 ± 0.00	100 ± 0.00	61.67 ± 5.57	16.67 ± 2.89	0.00 ± 0.00	0.00 ± 0.00	0.00 ± 0.00
360	100 ± 0.00	100 ± 0.00	23.33 ± 7.64	0.00 ± 0.00	0.00 ± 0.00	0.00 ± 0.00	0.00 ± 0.00
420	100 ± 0.00	100 ± 0.00	0.00 ± 0.00	0.00 ± 0.00	0.00 ± 0.00	0.00 ± 0.00	0.00 ± 0.00
480	100 ± 0.00	100 ± 0.00	0.00 ± 0.00	0.00 ± 0.00	0.00 ± 0.00	0.00 ± 0.00	0.00 ± 0.00
540	100 ± 0.00	100 ± 0.00	0.00 ± 0.00	0.00 ± 0.00	0.00 ± 0.00	0.00 ± 0.00	0.00 ± 0.00
600	100 ± 0.00	100 ± 0.00	0.00 ± 0.00	0.00 ± 0.00	0.00 ± 0.00	0.00 ± 0.00	0.00 ± 0.00

Values are expressed as means ± SDs (*n* = 3).

**Table 3:** The effect of clove extract on coral zooxanthellae, chlorophyll a, polyp length, and survival.

Time	Treatments (ppm)	Zooxanthellae (Cells × 10^7^ m^−2^)	Chlorophyll a (µg cm^−2^)	Polyp Length (cm/polyp)	Survival Rate (%)
10 min	C	5.10 ± 2.54 ^a^	45.72 ± 5.30 ^a^	1.05 ± 0.05 ^a^	100 ± 0.00 ^a^
	1500	5.05 ± 1.45 ^a^	44.21 ± 9.32 ^a^	1.03 ± 0.50 ^a^	100 ± 0.00 ^a^
	2500	5.02 ± 2.34 ^a^	47.07 ± 8.83 ^a^	0.65 ± 0.32 ^bc^	100 ± 0.00 ^a^
	5000	5.02 ± 1.45 ^a^	48.45 ± 7.05 ^a^	0.10 ± 0.01 ^c^	100 ± 0.00 ^a^
	7500	5.07 ± 1.36 ^a^	46.93 ± 9.32 ^a^	0.10 ± 0.01 ^c^	100 ± 0.00 ^a^
	10,000	5.06 ± 2.08 ^a^	47.21 ± 7.31 ^a^	0.10± 0.02 ^c^	100 ± 0.00 ^a^
24 h	C	5.12 ± 0.41 ^a^	46.03 ± 4.83 ^a^	1.05 ± 0.00 ^a^	100 ± 0.00 ^a^
	1500	4.53 ± 0.21 ^a^	43.02 ± 3.42 ^a^	1.03 ± 0.50 ^a^	90.00 ± 0.50 ^b^
	2500	3.21 ± 0.41 ^b^	32.33 ± 5.09 ^b^	0.20 ± 0.00 ^b^	85.00 ± 0.00 ^c^
	5000	2.92 ± 0.31 ^b^	24.04 ± 6.42 ^b^	0.10 ± 0.00 ^c^	0.00 ± 0.00 ^d^
	7500	0.04 ± 0.00 ^c^	0.90 ± 0.00 ^c^	0.00 ± 0.00 ^d^	0.00 ± 0.00 ^d^
	10,000	0.04 ± 0.00 ^c^	0.90 ± 0.00 ^c^	0.00 ± 0.00 ^d^	0.00 ± 0.00 ^d^

Different letters indicate significant differences among groups in Duncan’s multiple range test (*p* < 0.05). Values are expressed as means ± SDs (*n* = 30 colonies). C: control group.

**Table 4:** Clove extract for ciliate disease treatment evaluation.

Treatments (ppm)	Zooxanthellae (Cells×10^7^ m^−2^)	Chlorophyll a (µg cm^−2^)	Polyp Length (cm/polyp)	Survival Rate (%)
C	0.23 ± 0.15 ^b^	5.53 ± 0.12 ^c^	0.00 ± 0.00 ^e^	11.35 ± 5.73 ^b^
1500	5.03 ± 0.50 ^a^	50.21 ± 4.35 ^a^	1.00 ± 0.20 ^a^	100 ± 0.00 ^a^
2500	4.43 ± 0.42 ^a^	48.24 ± 2.55 ^a^	0.50 ± 0.21 ^b^	100 ± 0.00 ^a^
5000	4.56 ± 1.04 ^a^	43.00 ± 4.52 ^a^	0.20 ± 0.00 ^c^	100 ± 0.00 ^a^
7000	3.51 ± 1.21 ^ab^	32.25 ± 3.20 ^b^	0.10 ± 0.00 ^d^	100 ± 0.00 ^a^
10,000	3.23 ± 0.05 ^b^	30.02 ± 3.54 ^b^	0.10 ± 0.00 ^d^	100 ± 0.00 ^a^

Different letters indicate significant differences among groups in Duncan’s multiple range test (*p* < 0.05). Values are expressed as means ± SDs (*n* = 30 colonies). C: control group.

## Data Availability

Not applicable.
